# Dilated superficial penile dorsal vein in a child: Clinical images

**DOI:** 10.1002/ccr3.6043

**Published:** 2022-07-11

**Authors:** Alain Mwamba Mukendi, Gerald Tatenda Mataruka, Tshisola Miji Kasapato

**Affiliations:** ^1^ Division of Urology, Department of Surgery, Thelle Moroegane Regional Hospital University of the Witwatersrand Johannesburg South Africa

**Keywords:** childhood, dilated superficial vein, penile dorsal vein, penile vasculature, penis

## Abstract

Dilated superficial penile dorsal vein in childhood has never been reported. We present this index case as clinical image which after investigation no obvious cause was found. This could just be a normal anatomical variant or an idiopathic dilatation of the superficial penile dorsal vein.

## Graphical abstract

The incidence of dilated superficial penile dorsal vein in childhood is unknown, and the entity has never been reported. This is the first ever case illustrated as clinical image which could most likely be a normal anatomical variant or an idiopathic dilatation of the superficial penile dorsal vein.

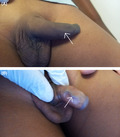

## 1 CASE PRESENTATION

**FIGURE 1:**
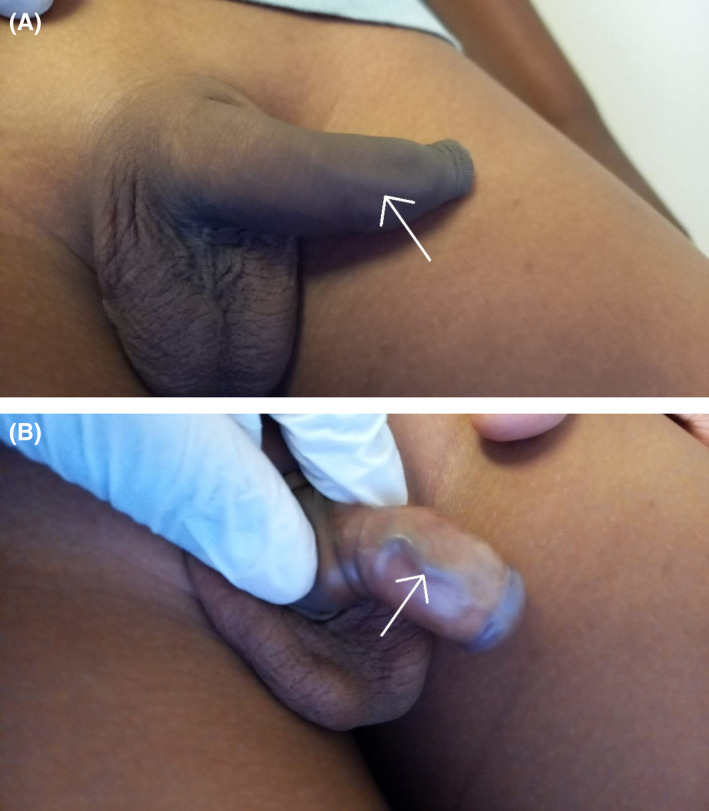
(A and B) Demonstrating a “cord like structure,” superficial dorsal penile vein shown by white arrow

**FIGURE 2:**
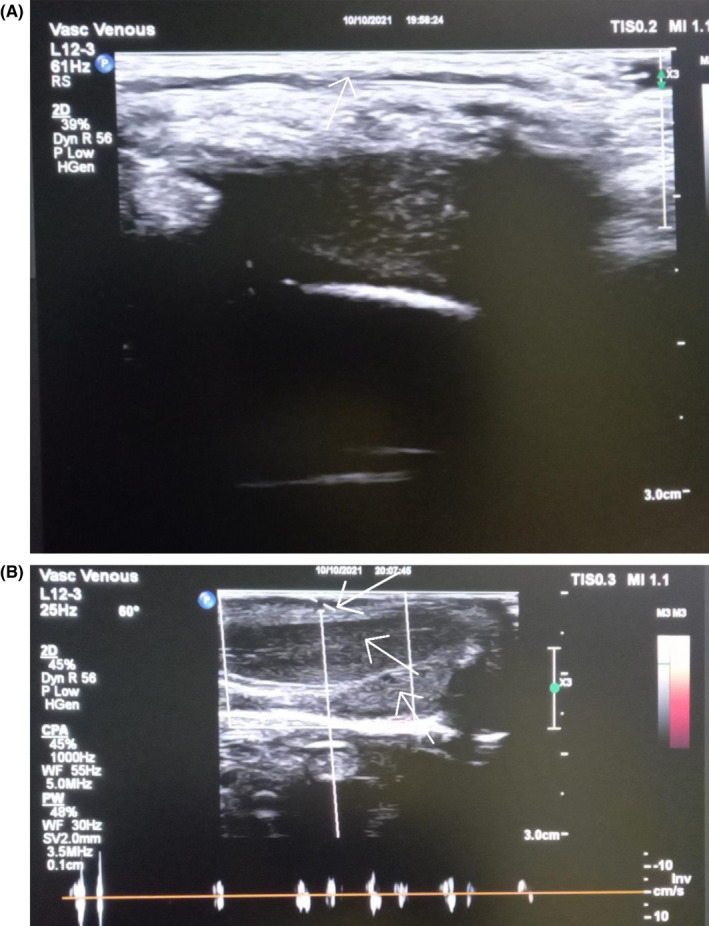
Demonstrating sonographic and Doppler images. (A) The white arrow pointing to the dilated superficial penile dorsal vein. (B) The upper white arrow pointing to the dilated superficial penile dorsal vein with evidence of venous flow and no thrombus; the middle arrow to the corpus cavernosum and the lower arrow to the corpus spongiosum

A 7 year‐old boy was brought in by his parents with about 3 weeks history of intermittent penile discomfort, which was not always related to micturition. No history of trauma, or recent instrumentation, or catheterization. On physical examination, he was uncircumcised and had a visible cord like structure on the dorsal aspect of his penis, which on foreskin retraction appeared to be a dilated superficial penile dorsal vein (SDV) and was non tender (Figure 1A,B). His stretched penile length was 4.9 cm. The rest of the examination was unremarkable. Uroflowmetry was normal. No abnormality was detected on urine microscopy and culture; and penile Doppler (Figure 2A,B) showed a dilated SDV with no thrombus.

## 2 DISCUSSION

In a pediatric population, penile pains/discomfort can result from primary or secondary causes. Primary causes include urinary tract infection, urethral stricture, meatal stenosis, phimosis, idiopathic penile arterial thrombosis, and trauma. Secondary causes (referred pains) resulting from dysfunctional elimination syndrome.^1^ History, physical examination, and urinalysis can help exclude most of the primary causes.

Dilated superficial penile dorsal vein associated with pains/discomfort is usually related to thrombophlebitis (mondor's disease), a rare entity seen in sexually active individuals where penile Doppler shows presence of a thrombus and absence of flow.^2^ We report the first case of a dilated superficial penile dorsal vein in grade schooler with absence of thrombus and presence of venous flow on Doppler. This could either be a normal anatomical variant or an idiopathic dilatation of the SDV.

## AUTHOR CONTRIBUTIONS

AMM conceived and designed the study, acquired the data, analyzed and interpreted the data, wrote the manuscript, and approved the final manuscript for publication. GTM acquired, analyzed and interpreted the data; approved final manuscript for publication. TMK acquired, analyzed and interpreted the data; obtained ethical approval, and approved final manuscript for publication.

## CONFLICT OF INTEREST

The authors have declared that they have no conflicts of interest to disclose.

## CONSENT

Written informed consent was obtained from parents for publication of this manuscript and accompanying pictures. A copy of the written consent is available for review by the Editor‐in‐Chief of this journal.

## Data Availability

The data that support the findings of this study are available from the corresponding author upon reasonable request.
